# Have health inequalities changed during childhood in the New Labour generation? Findings from the UK Millennium Cohort Study

**DOI:** 10.1136/bmjopen-2016-012868

**Published:** 2017-01-11

**Authors:** Emeline Rougeaux, Steven Hope, Catherine Law, Anna Pearce

**Affiliations:** Department of Population Policy and Practice, UCL Great Ormond Street Institute of Child Health, London, UK

**Keywords:** INEQUALITIES, CHILD HEALTH, LIFECOURSE EPIDEMIOLOGY, COHORT, POLICY

## Abstract

**Objectives:**

To examine how population-level socioeconomic health inequalities developed during childhood, for children born at the turn of the 21st century and who grew up with major initiatives to tackle health inequalities (under the New Labour Government).

**Setting:**

The UK.

**Participants:**

Singleton children in the Millennium Cohort Study at ages 3 (n=15 381), 5 (n=15 041), 7 (n=13 681) and 11 (n=13 112) years.

**Primary outcomes:**

Relative (prevalence ratios (PR)) and absolute health inequalities (prevalence differences (PD)) were estimated in longitudinal models by socioeconomic circumstances (SEC; using highest maternal *academic* attainment, ranging from ‘no academic qualifications’ to ‘degree’ (baseline)). Three health outcomes were examined: overweight (including obesity), limiting long-standing illness (LLSI), and socio-emotional difficulties (SED).

**Results:**

Relative and absolute inequalities in overweight, across the social gradient, emerged by age 5 and increased with age. By age 11, children with mothers who had no academic qualifications were considerably more likely to be overweight as compared with those with degree-educated mothers (PR=1.6 (95% CI 1.4 to 1.8), PD=12.9% (9.1% to 16.8%)). For LLSI, inequalities emerged by age 7 and remained at 11, but only for children whose mothers had no academic qualifications (PR=1.7 (1.3 to 2.3), PD=4.8% (2% to 7.5%)). Inequalities in SED (observed across the social gradient and at all ages) declined between 3 and 11, although remained large at 11 (eg, PR=2.4 (1.9 to 2.9), PD=13.4% (10.2% to 16.7%) comparing children whose mothers had no academic qualifications with those of degree-educated mothers).

**Conclusions:**

Although health inequalities have been well documented in cross-sectional and trend data in the UK, it is less clear how they develop during childhood. We found that relative and absolute health inequalities persisted, and in some cases widened, for a cohort of children born at the turn of the century. Further research examining and comparing the pathways through which SECs influence health may further our understanding of how inequalities could be prevented in future generations of children.

**Strengths and limitations of this study**

- This is the first study to examine how population-level inequalities in health developed during childhood in a UK cohort who were born in 2000–2002 and grew up in the context of unprecedented initiatives to reduce health inequalities (under the New Labour Government).
- Evaluation of New Labour's policies was, however, not possible as we cannot assess what would have happened in their absence.
- We used data from a large nationally representative sample of UK children, which includes a range of health, demographic and socioeconomic data recorded throughout childhood.
- We carried out longitudinal analyses of relative and absolute inequalities for three important physical and mental health outcomes (overweight, limiting long-standing illness and socio-emotional difficulties), assessed across the socioeconomic gradient, measured using maternal education and income.
- Response weights were used to account for attrition, and sensitivity analyses indicated that item missingness was unlikely to have biased the results.

## Introduction

Children from less advantaged backgrounds have, on average, worse health than their more advantaged peers. This fuels inequalities in subsequent life chances (such as educational achievement and employment opportunities) and health and well-being in adulthood.^1–3^ Socioeconomic inequalities in health are unfair and avoidable, yet research indicates that inequalities for children and young people may have widened since the 1980s for many aspects of health and health behaviours, including overweight,^4^ physical activity, psychological and physical well-being.^5–8^ However, the majority of research has documented inequalities in children at single points in time. Although there is evidence of a possible period of equalisation during adolescence,^8^ this has largely been based on cross-sectional data and much less is known about how population-level health inequalities change in the same group of children as they age throughout childhood. Cohort data would improve our understanding of how health inequalities develop over this important period of the life course and whether patterns vary for different aspects of health.

At the start of the New Labour Government (1997–2010), a pledge to eradicate child poverty in a generation^9^ and the introduction of a strategy to sustainably tackle inequalities in health,^10^ led to a number of policies to tackle the social determinants of health, with a particular focus on the early years (such as Sure Start Children's Centres and increases in statutory paid parental leave^9–11^). Although it would be impossible to assess what would have happened to health inequalities in the absence of these policies, it is important to track how inequalities changed for the children who grew up during this period of concerted policy efforts. This could help to inform future policies and practice, by highlighting the aspects of health or periods in childhood that might benefit from greater focus.

The aim of this study was to examine how population-level socioeconomic inequalities in health developed throughout childhood for those born at the beginning of the 21st century. Three health measures were assessed across the socioeconomic gradient: overweight, limiting long-standing illness (LLSI) and socio-emotional difficulty (SED). These are prevalent physical and mental health outcomes which may significantly impact current and future health and well-being.^1–3^

## Methods

### Sample

We used data from the UK Millennium Cohort Study (MCS), a nationally representative survey of children born in the UK, in September 2000 to January 2002. A stratified clustered sampling design was used to oversample children living in Wales, Scotland and Northern Ireland, disadvantaged areas and those with high proportions of ethnic minority groups (in England).^12^ The parents of cohort children were first contacted for interview at 9 months, when information was collected on 72% of those contacted, providing information for 18 818 children (of which 18 296 were singletons and are the focus of this paper). Children were followed up at 3, 5, 7 and 11 years of age and 68% (n=13 112) of singletons took part in the age 11 interview.^12–16^ Interviews were carried out in the home with the main respondent, predominantly the natural mother, and if applicable, the partner (where possible).^12^

### Health outcomes

Dichotomous measures were constructed at ages 3, 5, 7 and 11 years for the following three outcomes:

- *Overweight (including obesity):* Children's height and weight were measured by interviewers (using Tanita BF-522W scales for weight and a Leceister statiometer for height^17^). Body Mass Index (BMI; kg/m^2^) was categorised into being overweight (including obesity) or of healthy weight using the International Obesity Task Force (IOTF) age and sex adjusted cut-offs for children.^18^
- *Limiting long-standing illness* (LLSI): Main respondents were asked if their child had any long-standing illness (physical or mental health conditions or illnesses lasting or expected to last 12 months or more) that limited the child in their everyday activities. Children were classified as having LLSI or not.
- *Socio-emotional difficulty (SED)*: The Strengths and Difficulties Questionnaire (SDQ)^19^ was completed by the main respondent. The ‘total difficulties score’ is the sum of four subscales of the SDQ which capture key areas of child socio-emotional well-being: emotional symptoms, conduct problems, hyperactivity and peer problems. Children were classified with validated cut-offs,^19^ as having SED (borderline/abnormal score, 14–40) or having no SED (normal score, 0–13). Where one or two (out of a total of five) items were missing in a subscale of the total difficulties score, values were imputed based on the mean of other item responses.^20^

### Measure of socioeconomic circumstances

Socioeconomic circumstances (SECs) were represented by natural mother's highest *academic* attainment (hereafter referred to as ‘maternal education’) when the cohort member was aged 3, 5, 7 and 11 years and categorised as: degree, diploma (in higher education—shortened to ‘diploma’ hereafter), General Certificate of Education Advanced Level (A level), General Certificate of Secondary Education (GCSE) grades A*–C, GCSE grades D–G or no academic qualifications (shortened to ‘no qualifications’ hereafter). Those with ‘other’ maternal qualifications are shown in table 1 but were excluded thereafter. Maternal education was used as the main measure of SECs in the analyses because it was stable throughout the period under study, is frequently used to assess inequalities in children,^21^ and had limited missing data (<1%). Furthermore, this measure can be applied to mothers who have never had paid employment. As a sensitivity analysis, analyses were repeated using an alternative measure of SECs (quintiles of equivalised household income) and patterns of results were replicated (data not shown).

**Table 1:** Cross-sectional socio-demographic characteristics and health outcomes of singletons in the Millennium Cohort Study at ages 3 (n=15 381) and 11 (n=13 112); number (n), weighted percentages (%)

	Age 3					Age 11				
	Total		Overweight (n=13 315) %	Limiting long-standing illness (n=15 232) %	Socio-emotional difficulty (n=14 217) %	Total		Overweight (n=11 790) %	Limiting long-standing illness (n=13 002) %	Socio-emotional difficulty (n=12 584) %
	n	%				n	%			
Cohort member's sex										
Male	7862	51.0	23.1	3.3	23.7	6632	51.7	26.6	9.9	20.7
Female	7519	49.1	24.9	2.9	18.5	6480	48.3	31.3	6.5	13.6
χ^2^ test p value	–	–	0.04	0.2	<0.001	–	–	<0.001	<0.001	<0.001
Cohort member's ethnic group										
White	12 768	86.5	24.1	3.0	20.3	10 837	84.4	27.7	8.4	17.3
Indian	398	1.9	12.4	3.8	25.0	338	2.1	30.1	5.2	17.1
Pakistani/Bangladeshi	1024	4.3	22.1	3.7	39.5	943	5.1	37.7	6.1	14.8
Black/Black British	505	2.8	29.7	2.7	20.9	423	3.5	40.7	6.0	13.6
Mixed	444	3.2	24.1	5.4	23.6	378	3.5	34.9	12.6	20.6
Other (incl. Chinese)	223	1.3	24.3	3.2	28.9	186	1.5	26.1	4.3	17.2
χ^2^ test p value	–	–	0.002	0.08	<0.001	–	–	<0.001	0.01	0.4
Maternal age at first live birth (years)										
12–17	979	6.5	25.1	4.9	38.6	782	8.2	26.2	8.9	28.3
18–20	2661	18.2	24.3	4.1	31.7	2251	21.4	32.6	10.3	24.5
21–25	3769	25.1	25.0	3.0	22.1	3214	25.4	30.3	8.5	18.0
26–30	4118	29.0	23.9	2.4	14.7	3622	27.9	27.2	7.3	11.7
31 or more	2711	20.2	22.5	2.7	12.0	2370	17.2	25.0	6.3	9.8
χ^2^ test p value	–	–	0.5	<0.001	<0.001	–	–	<0.001	0.002	<0.001
Maternal highest academic attainment										
Degree	2655	17.8	22.6	2.8	8.8	2936	19.0	21.2	6.6	8.2
Diploma	1450	9.7	23.1	2.3	13.7	1589	11.6	26.1	6.0	11.1
A levels	1465	9.5	20.8	1.9	13.1	1137	8.0	28.0	7.3	13.8
GCSE A*–C	4986	34.0	24.1	3.5	20.9	3828	30.9	30.5	8.9	17.8
GCSE D–G	1597	11.0	26.6	3.2	31.2	1200	10.8	32.5	8.6	23.6
Other (incl. overseas)	619	3.6	26.9	4.0	32.5	832	6.9	32.9	8.8	23.8
No qualifications	2499	14.5	26.1	3.8	40.0	1521	12.9	34.5	11.3	28.6
χ^2^ test p value	–	–	0.03	0.04	<0.001	–	–	<0.001	<0.001	<0.001

General Certificate of Education Advanced Level, A level; GCSE,General Certificate of Secondary Education.

### Covariates

We adjusted for cohort member's sex and ethnicity, and maternal age at first live birth (as this was found to be associated with the health outcomes elsewhere and might also influence maternal education^22^ ^23^) (categories shown in table 1). In general, these variables were significantly associated with the three health outcomes (as shown in table 1 for the earliest (age 3) and latest (age 11) sweeps only).

### Analysis

Population-averaged inequalities in each health outcome were estimated during childhood using generalised estimating equations (GEE) for panel data, taking into account the correlation of repeated measurements from the same children. Poisson regression models were used to estimate relative inequalities (given by prevalence ratios (PRs)) and absolute inequalities (given by prevalence differences (PDs)); these compare the prevalence in each maternal education category to baseline (degree), either as a ratio (PR) or a difference (PD).

An interaction term between maternal education and age was included in the model in order to estimate PDs and PRs at age 3, 5, 7 and 11 years. The 95% CIs for the PDs and PRs represent statistical certainty for the age-specific inequalities (using degree as baseline at a given age). Probability values (p values) derived from the interaction term between maternal education and age indicate whether PRs and PDs at ages 5, 7 and 11 were statistically significantly different from those at age 3 (baseline). These p values are indicated in the results tables with ‡(p≤0.05) and §(p≤0.001).

Analyses were carried out before and after adjusting for covariates, adjusted results are the focus of the paper, with unadjusted results provided in online supplementary annex I. The analytic sample comprised singleton children who had data on the covariates (recorded at age 9 months) and relevant health outcomes for at least one of the relevant time points (see tables 2–4 for sample numbers and missing data). Weights were used to account for survey design and attrition to the most recent completed interview. As a sensitivity analysis, we repeated the models without weights and the patterns of inequality over time were unchanged. We also carried out multiple imputations on each of the health outcomes as a sensitivity analysis to assess bias from item missingness and the patterns of inequality over time remained unchanged. As a final sensitivity analysis, the models were repeated in a sample limited to cases where the main respondent was always the natural mother (to ensure that any changes in health inequalities were not the result of changes in main respondent) and results were unchanged. Analyses were carried out in Stata/SE 13.1 (StataCorp LP, Texas, USA). Data were downloaded from the UK Data Service, University of Essex and University of Manchester, in March 2014.

**Table 2:** Socioeconomic inequalities in overweight in the Millennium Cohort Study by maternal academic attainment at ages 3, 5, 7 and 11 (n=14 872; 46 094 observations)

	Age 3	Age 5	Age 7	Age 11
Relative inequality: adjusted† prevalence ratios (PR) for overweight (95% CIs)				
Degree	–	–	–	–
Diploma	1.0 (0.9 to 1.1)	1.2 (1.0 to 1.4)‡	1.2 (1.0 to 1.4)	1.2 (1.1 to 1.4)‡
A Level	0.9 (0.8 to 1.0)	1.0 (0.9 to 1.2)	1.2 (1.0 to 1.4)‡	1.2 (1.1 to 1.4)§
GCSE A*–C	1.0 (0.9 to 1.2)	1.2 (1.1 to 1.4)‡	1.4 (1.2 to 1.5)§	1.5 (1.3 to 1.6)§
GCSE D–G	1.1 (1.0 to 1.3)	1.3 (1.1 to 1.5)	1.4 (1.2 to 1.6)‡	1.5 (1.3 to 1.7)‡
No qualifications	1.1 (0.9 to 1.2)	1.3 (1.1 to 1.5)‡	1.5 (1.3 to 1.8)§	1.6 (1.4 to 1.8)§
Absolute inequality: adjusted† prevalence differences (PD) for overweight (95% CIs)				
Degree	–	–	–	–
Diploma	−0.2 (−3.4 to 3.0)	3.2 (0.3 to 6.1)‡	2.6 (-0.1 to 5.3)	5 (1.9 to 8.0)‡
A Level	−2.8 (−6.0 to 0.3)	0.5 (-2.5 to 3.5)‡	2.7 (-0.2 to 5.8)‡	5.2 (1.7 to 8.7)§
GCSE A*–C	0.9 (−1.6 to 3.4)	4.2 (2.0 to 6.4)‡	5.9 (3.7 to 8.1)§	9.6 (7.1 to 12.1)§
GCSE D–G	3.2 (−0.3 to 6.8)	5.1 (1.8 to 8.3)	6 (2.8 to 9.2)	10.3 (6.5 to 14)‡
No qualifications	1.8 (−1.6 to 5.2)	5.8 (2.7 to 8.9)‡	8.3 (5.2 to 11.5)§	12.9 (9.1 to 16.8)§

Missing data (n) at age 3, 5, 7, 11 for: overweight: 1373, 251, 340, 410; maternal academic attainment: 110, 100, 88, 69; missing data (n) for maternal age at first live birth: 1359; cohort member ethnicity: 31.

†Adjusted for maternal age at first live birth, child sex and ethnicity.

‡≤0.05, §≤0.001; significance test p-value for age PR differences (interaction) and PD differences (pairwise comparisons) (age 3 baseline).

General Certificate of Education Advanced Level, A level; GCSE,General Certificate of Secondary Education.

**Table 3:** Socioeconomic inequalities in limiting long-standing illness (LLSI) in the Millennium Cohort Study by maternal academic attainment at ages 3, 5, 7 and 11 (n=15 250; 50 401 observations)

	Age 3	Age 5	Age 7	Age 11
Relative inequality: adjusted† prevalence ratios (PR) for LLSI (95% CIs)				
Degree	–	–	–	–
Diploma	0.8 (0.5 to 1.2)	0.7 (0.5 to 1.0)	1.1 (0.8 to 1.4)	0.9 (0.7 to 1.2)
A Level	0.6 (0.4 to 1.0)	0.9 (0.7 to 1.3)	1.1 (0.8 to 1.5)‡	1.1 (0.8 to 1.5)‡
GCSE A*–C	1.1 (0.8 to 1.5)	1.1 (0.9 to 1.4)	1.3 (1.0 to 1.6)	1.3 (1.0 to 1.6)
GCSE D–G	0.8 (0.5 to 1.3)	1.1 (0.9 to 1.6)	1.4 (1.0 to 1.9)	1.1 (0.8 to 1.5)
No qualifications	1.1 (0.8 to 1.6)	1.2 (0.9 to 1.6)	1.7 (1.3 to 2.3)‡	1.7 (1.3 to 2.3)‡
Absolute inequality: adjusted† prevalence differences (PD) for LLSI (95% CIs)				
Degree	–	–	–	–
Diploma	−0.8 (−2.0 to 0.4)	−1.7 (−3.2 to 0.1)	0.3 (−1.4 to 2.0)	−0.4 (−2.2 to 1.4)
A Level	−1.4 (−2.5 to −0.2)	-0.5 (-2.2 to 1.3)	0.6 (−1.2 to 2.5)‡	0.8 (−1.4 to 3.0)
GCSE A*–C	0.4 (−0.7 to 1.5)	0.6 (−0.8 to 2.0)	1.3 (−0.1 to 2.7)	1.8 (0.2 to 3.4)
GCSE D–G	−0.5 (−1.9 to 0.9)	0.3 (−1.7 to 2.3)	2 (−0.3 to 4.2)‡	0.9 (−1.3 to 3.1)
No qualifications	0.5 (−0.9 to 1.8)	1.3 (−0.5 to 3.1)	3.9 (1.8 to 6.0)§	4.8 (2.0 to 7.5)‡

Missing data (n) at age 3, 5, 7, 11 for: LLSI: 149, 86, 82, 110; maternal academic attainment: 110, 100, 88, 69; missing data (n) for maternal age at first live birth: 1359; cohort member ethnicity: 31.

†Adjusted for maternal age at first live birth, child sex and ethnicity.

‡≤0.05, §≤0.001; significance test p-value for age PR differences (interaction) and PD differences (pairwise comparisons) (age 3 baseline).

General Certificate of Education Advanced Level, A level; GCSE,General Certificate of Secondary Education.

**Table 4:** Socioeconomic inequalities in socio-emotional difficulty (SED) in the Millennium Cohort Study by maternal academic attainment at ages 3, 5, 7 and 11 (n=15 103; 48 832 observations)

	Age 3	Age 5	Age 7	Age 11
Relative inequality: adjusted† prevalence ratios (PR) for SED (95% CIs)				
Degree		–	–	–
Diploma	1.5 (1.2 to 1.8)	1.5 (1.1 to 1.9)	1.6 (1.3 to 2.0)	1.3 (1.0 to 1.6)
A Level	1.3 (1.1 to 1.6)	1.8 (1.4 to 2.4)‡	1.5 (1.1 to 1.9)	1.4 (1.1 to 1.8)
GCSE A*–C	1.9 (1.6 to 2.3)	1.9 (1.5 to 2.4)	1.9 (1.6 to 2.3)	1.7 (1.5 to 2.1)
GCSE D–G	2.7 (2.3 to 3.2)	2.9 (2.2 to 3.7)	2.5 (2.0 to 3.1)	2.1 (1.7 to 2.5)‡
No qualifications	3.1 (2.6 to 3.7)	4 (3.2 to 5.1)‡	3.1 (2.5 to 3.8)	2.4 (1.9 to 2.9)‡
Absolute inequality: adjusted† prevalence differences (PD) (95% CIs)				
Degree		–	–	–
Diploma	5.5 (2.6 to 8.4)	2.4 (0.5 to 4.3)‡	4.5 (2.3 to 6.7)	2.7 (0.3 to 5.0)
A Level	3.4 (0.7 to 6.2)	4.4 (2.3 to 6.6)	3.3 (0.9 to 5.7)	4.2 (1.2 to 7.1)
GCSE A*–C	10.3 (8.1 to 12.4)	4.8 (3.4 to 6.3)§	6.6 (4.8 to 8.4)‡	7.4 (5.4 to 9.4)‡
GCSE D–G	18.8 (15.7 to 22.0)	9.8 (7.4 to 12.2)§	10.8 (8.0 to 13.6)§	10.9 (7.7 to 14.0)§
No qualifications	23.1 (19.9 to 26.3)	16.1 (13.5 to 18.7)§	15.8 (12.9 to 18.7)§	13.4 (10.2 to 16.7)§

Missing data (n) at age 3, 5, 7, 11 for: SED: 1164, 647,492 528; maternal academic attainment: 110, 100, 88, 69; missing data (n) for maternal age at first live birth: 1359; cohort member ethnicity: 31.

†Adjusted for maternal age at first live birth, child sex and ethnicity.

‡≤0.05, §≤0.001; significance test p value for age PR differences (interaction) and PD differences (pairwise comparisons) (age 3 baseline).

General Certificate of Education Advanced Level, A level; GCSE,General Certificate of Secondary Education.

10.1136/bmjopen-2016-012868.supp1 supplementary annex

## Results

### Overweight (including obesity)

**Figure 1:**
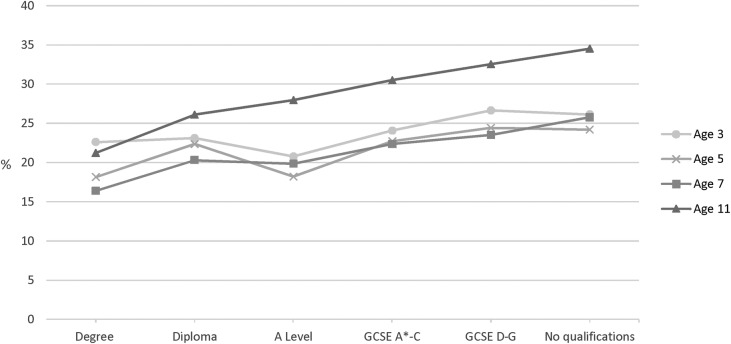
Prevalence of overweight in singletons in the Millennium Cohort Study at ages 3 (n=15 381), 5 (n=15 041), 7 (n=13 681) and 11 (n=13 112) by concurrent maternal academic attainment, weighted %. GCSE,General Certificate of Secondary Education.

At age 3, 24.0% (95% CI 23.1% to 24.9%) of children were overweight, falling slightly to 21.9% (21.1% to 22.7%) and 21.4% (20.6% to 22.2%) at ages 5 and 7 respectively, before increasing to 28.9% (27.9% to 29.9%) at age 11. A visible social gradient in overweight had emerged by age 7, which then steepened at age 11 (figure 1; table 1 shows numbers and percentages at ages 3 and 11).

Table 2 indicates that small relative and absolute inequalities in overweight (after adjusting for covariates) emerged by age 5 and were observed across the socioeconomic gradient (using ‘degree’ as baseline) (table 2). PRs and PDs increased with age, and by 11 years children whose mothers had no qualifications were 60% (PR: 1.6; 95% CI 1.4 to 1.8) more likely to be overweight than children of degree-educated mothers, and the absolute difference in prevalence was 12.9% (9.1% to 16.8%). A statistically significant interaction (between age and maternal education) confirmed a widening of absolute and relative inequalities, across the gradient, over time (table 2). Patterns were similar for unadjusted analyses (see online supplementary annex I).

### Limiting long-standing illness

**Figure 2:**
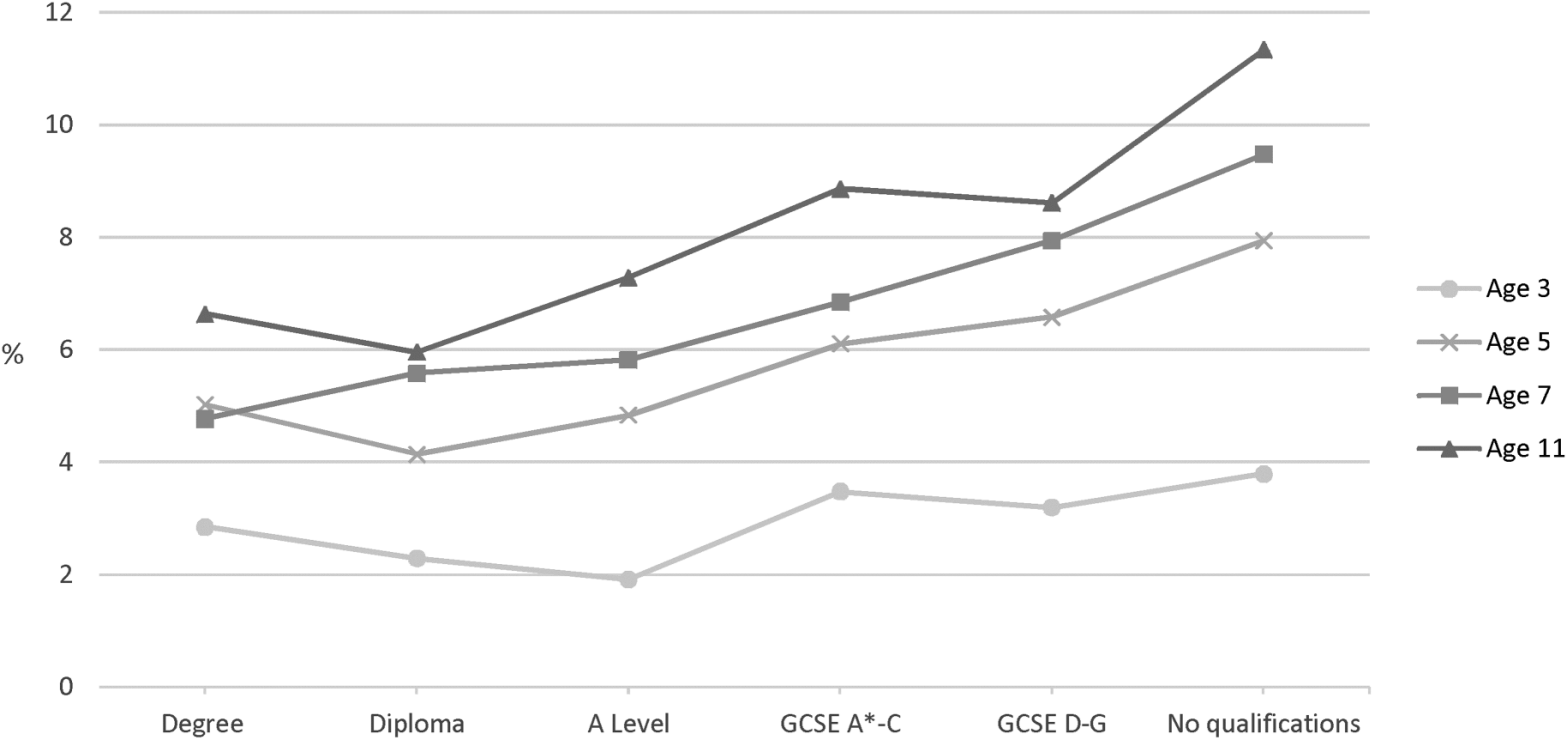
Prevalence of LLSI in singletons in the Millennium Cohort Study at ages 3 (n=15 381), 5 (n=15 041), 7 (n=13 681) and 11 (n=13 112) by concurrent maternal academic attainment, weighted %. GCSE,General Certificate of Secondary Education; LLSI, limiting long-standing illness.

The prevalence of LLSI increased with age from 3.1% (95% CI 2.8% to 3.5%) at age 3, to 5.9% (5.5% to 6.4%), 6.8% (6.3% to 7.4%) and then 8.3% (7.7% to 8.9%) at ages 5, 7 and 11 respectively. A gradient in the prevalence of LLSI by maternal education appeared from age 5 and steepened slightly up to age 11(figure 2; table 1 shows numbers and percentages at ages 3 and 11).

Relative and absolute inequalities (after adjusting for covariates) were only observed from age 7 and were limited to children with mothers who had no qualifications (table 3). By age 11, children whose mothers had no qualifications were 70% (PR: 1.7; 95% CI 1.3 to 2.3) more likely to have LLSI (compared with those whose mothers had a degree), and the absolute difference in prevalence was 4.8% (95% CI 2.0% to 7.5%). The interaction term confirms that inequalities between children whose mothers had no qualifications compared with those with a degree-educated mothers were significantly greater at age 7 and 11 than at age 3 (table 3). The unadjusted analyses shows similar patterns (see online supplementary annex I).

### Socio-emotional difficulty

**Figure 3:**
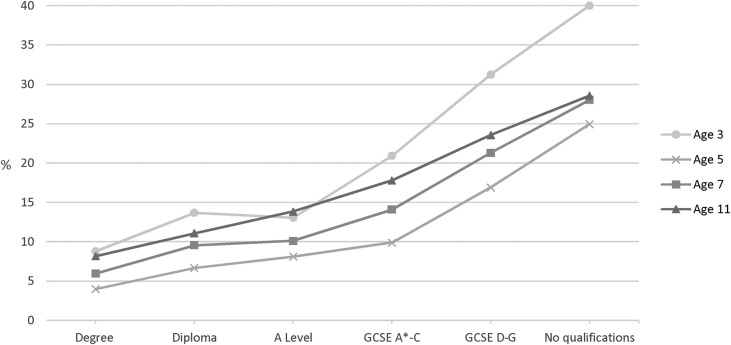
Prevalence of SED in the Millennium Cohort Study in singletons at ages 3 (n=15 381), 5 (n=15 041), 7 (n=13 681) and 11 (n=13 112) by concurrent maternal academic attainment, weighted %. GCSE,General Certificate of Secondary Education; SED, socio-emotional difficulties.

At age 3, 21.2% (20% to 22.4%) of children were classified as having socio-emotional difficulty (SED); this declined to 11.4% (10.6% to 12.1%) at age 5 but increased to 14.7% (13.8% to 15.6%) and 17.2% (16.2% to 18.3%) at ages 7 and 11 respectively. There was a strong gradient in the prevalence of SED by maternal education at age 3. The gradient was less steep but remained at ages 5, 7 and 11 (figure 3; table 1 shows numbers and percentages at ages 3 and 11).

Large relative and absolute inequalities in SED (after adjusting for covariates) were observed across the socioeconomic gradient from the age of 3. Relative inequalities appeared to increase at age 5, and then decrease thereafter (table 4). The interaction term between age and maternal education indicates that compared with age 3, relative inequalities (between the highest and lowest socioeconomic groups) had significantly increased by age 5, but by age 11 had become significantly smaller. In contrast, absolute inequalities declined steadily (and significantly) after age 3. This apparent discrepancy between absolute and relative inequalities was largely driven by the reduction in the prevalence of SED (overall and in every socioeconomic group) after age 3. Despite this, inequalities at age 11 remained; children whose mothers had no qualifications were more than twice as likely to have SED (PR: 2.4 (1.9% to 2.9%)), with an absolute adjusted difference of 13.4% (10.2% to 16.7%). In the unadjusted analyses inequalities at each age were greater, but patterns of change over age remained the same (see online supplementary annex I).

## Discussion

### Summary of findings

Health inequalities persisted and in some cases widened in a representative contemporary cohort of UK children who grew up during a period of major policy initiatives designed to address health inequalities. However, patterns of inequality over time varied by health outcome. The socioeconomic gradient in overweight increased steadily during childhood, in relative and absolute terms. In LLSI, inequality also widened between ages 3 and 11 years. However, the inequalities in LLSI were not seen across the entire socioeconomic gradient and were confined to the most disadvantaged group (children with mothers with no academic qualifications). In contrast, inequalities in SED, which were seen across the entire gradient, decreased in relative and absolute terms by age 11, but nevertheless remained substantial.

### Strengths and limitations

This is the first study to document how population-level health inequalities have changed during childhood in a nationally representative cohort of UK children born at the beginning of the 21st century. Population average models were used to account for the longitudinal study design and correlation of repeated measurements, and an interaction term between maternal education (our socioeconomic measure) and age was included in order to examine whether differences in health inequalities by age were statistically significant. The range of social and health information available in the MCS allowed us to examine three important outcomes (indicating physical and mental health) and two measures of SECs (maternal education and household income). We have provided estimates of relative and absolute inequality, as recommended for inequalities research^24^ ^25^ and an approach also used when monitoring progress towards national inequalities targets.^10^ Overweight was based on measured heights and weights and classified using validated cut-offs.^18^ Questions regarding LLSI are widely used in other health and social surveys^26^ and SED was assessed using the SDQ, which is a validated tool for measuring socio-emotional difficulty in children.^27^ ^28^ The content of the SDQ is consistent throughout childhood with the exception of three items which have been altered for preschool children (ages 2–4) to make them more age appropriate (eg, ‘often lies or cheats’ has been replaced by ‘often argumentative with adults’). The preschool and regular versions of the SDQ have been validated^27^ ^28^ and it is unlikely the item changes would have affected our results.

LLSI and SED were rated by the main respondent (usually the mother) and thus reflected the parent's perceptions, which may be influenced by socio-demographic characteristics, personal opinions, their own experiences or the context in which they observe the child.^29^ ^30^ Loss to follow-up is a problem common to all cohort studies, and the percentage of attrition in the MCS increased at every data collection time point so that, by age 11, 31% of the original cohort did not take part. While response weights were used to account for attrition, they do not overcome any bias due to item missingness. Item missingness differed by age, thus possibly biasing our estimates of change in health inequality over time. To assess this we repeated our analyses in a complete case sample (i.e children who had health outcomes at all four sweeps) and after running multiple imputations on the health outcomes found little change in the pattern of results for either. Finally, it was not possible to evaluate the impacts of policies with which the children of the MCS grew up as we could not assess what would have happened in their absence.

### Comparison with existing literature

Few studies have examined how health inequalities develop during childhood. To the best of our knowledge, the only other recent cohort (other than the MCS) that allows tracking of national-level inequality in UK children growing up in the 2000s is the Growing Up in Scotland (GUS) Study. Analyses using data from the first GUS birth cohort (born 2004/2005) show some similar findings for Scotland to those observed in the MCS (across the UK). For example, inequalities in obesity emerged at age 6 and widened by age 8^31^ and there were inequalities in socio-emotional difficulty at age 4^32^ which appeared to persist to age 7.^33^ An analysis of the Avon Longitudinal Study of Parents and their Children (ALSPAC) found that inequalities in BMI emerged at age 4 and increased to late childhood.^34^ More research on the same cohort found that lower maternal qualification was associated with lower height, greater adiposity, higher blood pressure and higher SED; over time (to age 11) inequalities increased in height and decreased for blood pressure, while those in fat mass and SED remained the same.^35^ However, ALSPAC is not representative of the UK population, and these children were born in the early 1990s (and therefore the context in which they were growing up was likely to have been different to the cohort that we studied). Research has suggested that early adolescence may be a period of equalisation for some health outcomes (although inequalities may re-emerge in adulthood).^36^ Our analysis showed little indication of equalisation by age 11. The only reduction in inequality was found for SED, although social differences remained substantial at 11 years. Recent research highlighted previously has indicated that equalisation may be shifting to later adolescence.^8^ This should be further assessed as the MCS participants enter mid and late adolescence.

### Implications for policy and practice

For the MCS children, indicators of inequalities in physical and mental health emerged early in life and persisted throughout childhood. This was despite growing up during unprecedented policy efforts to tackle health inequalities, which included interventions to improve incomes and employment in disadvantaged families as well as factors linked to child health such as neighbourhoods, housing, childcare and maternal health-related behaviours.^10^ The reasons why New Labour's concerted policy efforts to reduce inequalities in child health were only partly successful continue to be the subject of debate.^9^ ^37^ Explanations have included lack of understanding of the mechanisms through which SECs and child health are linked, insufficient focus on those most in need and on inequalities in income, and inadequate scale and timescale for implemented interventions.^37^ Earlier MCS analyses have highlighted potential mechanisms that may underlie cross-sectional inequalities in children's health, which range from parents' and children's health behaviours to parenting and the home environment.^38–42^ Further research examining and comparing the pathways through which health inequalities develop throughout childhood may further our understanding of how they might be alleviated. Evaluations of existing interventions to reduce child health inequalities are also needed to understand their effect on health inequalities over the lifecourse. For the MCS children and their contemporaries, adolescence may offer a second opportunity to reduce health inequalities.^43^
