# Supplementary material for: Have health inequalities changed during childhood in the New Labour generation? Findings from the UK Millennium Cohort Study
Source: BMJ Open. 2017 Jan 11;7(1):e012868. doi: 10.1136/bmjopen-2016-012868 (PMC5253527; doi:10.1136/bmjopen-2016-012868)
Supplement: supplementary annex [file bmjopen-2016-012868supp_annex.pdf]

Annex I. Socio-economic inequalities in the Millennium Cohort Study by maternal academic attainment at ages 3, 5, 7 and 11; prevalence ratios (PR) & prevalence difference (PD) (95% confidence intervals [CI])- Unadjusted results

	Overweight (n=14872; 46094 observations)				Limiting Long-Standing Illness (n=15401; 50401 observations)				Socio-emotional Difficulty (n=15103; 48832 observations)			
	Age 3	Age 5	Age 7	Age 11	Age 3	Age 5	Age 7	Age 11	Age 3	Age 5	Age 7	Age 11
Relative inequality: PR (95%CI)												
Degree	-	-	-		-	-	-		-	-	-	
Diploma	1.0 (0.9;1.2)	1.2 (1.0;1.4) [i]	1.2 (1.0;1.4)	1.2 (1.1;1.4) [i]	0.8 (0.5;1.2)	0.7 (0.5;1.0)	1.1 (0.8;1.5)	1.0 (0.8;1.3)	1.7 (1.4;2.0)	1.6 (1.2;2.1)	1.8 (1.4;2.2)	1.4 (1.2;1.8)
A Level	0.9 (0.8;1.0)	1.0 (0.9;1.2)	1.2 (1.0;1.4) [i]	1.2 (1.1;1.4) [ii]	0.6 (0.4;1.0)	1.0 (0.7;1.3)	1.2 (0.9;1.6) [i]	1.2 (0.9;1.6) [i]	1.5 (1.2;1.8)	2.1 (1.6;2.7) [i]	1.6 (1.3;2.1)	1.6 (1.3;2.0)
GCSE A*-C	1.0 (0.9;1.2)	1.2 (1.1;1.4) [i]	1.4 (1.2;1.5) [ii]	1.4 (1.3;1.6) [ii]	1.2 (0.9;1.7)	1.2 (1.0;1.5)	1.4 (1.1;1.7)	1.4 (1.1;1.7)	2.4 (2.0-2.8)	2.3 (1.9;2.9)	2.3 (1.9;2.8)	2.1 (1.8;2.5)
GCSE D-G	1.1 (1.0;1.3)	1.3 (1.1;1.5)	1.4 (1.2;1.6) [i]	1.5 (1.3;1.7) [i]	1.0 (0.6;1.5)	1.2 (0.9;1.6)	1.5 (1.1;2.1)	1.3 (1.0;1.7)	3.6 (3.0;4.2)	3.7 (2.9;4.8)	3.2 (2.6;4.0)	2.7 (2.2;3.3) [i]
No qualifications	1.1 (1.0;1.2)	1.3 (1.1;1.5) [i]	1.5 (1.3;1.7) [ii]	1.6 (1.4;1.8) [ii]	1.3 (0.9;1.9)	1.4 (1.1;1.9)	2.0 (1.6;2.6) [i]	2 (1.5;2.6)	4.5 (3.8;5.2)	5.7 (4.6;7.2) [i]	4.4 (3.6;5.4)	3.3 (2.7;4.0) [i]
Absolute inequality: PD (95%CI)												
Degree	-	-	-		-	-	-		-	-	-	
Diploma	-0.04 (-3.2;3.2)	3.3 (0.4;6.3) [i]	2.7 (-0.1;5.4)	5.1 (2.0;8.2) [i]	-0.7 (-1.8;0.5)	-1.4 (-2.9;0.1)	0.5 (-1.1;2.1)	-0.1 (-1.8;1.6)	6.0 (3.4;8.6)	2.7 (1.0;4.4) [i]	4.9 (2.9;6.9)	3.6 (1.4;5.8)
A Level	-2.9 (-6.1; 0.2)	0.4 (-2.6;3.4) [i]	2.7 (-0.3;5.7) [i]	5.0 (1.5;8.5) [ii]	-1.2 (-2.3;-0.1)	-0.2 (-1.8;1.5)	0.9 (-0.9;2.6) [i]	1.1 (-1.1;3.2) [i]	4.5 (2.0;6.9)	4.8 (2.8;6.8)	3.9 (1.7;6.2)	5.0 (2.3;7.7)
GCSE A*-C	0.8 (-1.7;3.2)	4 (1.9;6.2) [i]	5.8 (3.6;8.7) [ii]	9.4 (6.9;11.8) [ii]	0.7 (-0.3;1.7)	1.1 (-0.2;2.4)	1.8 (0.5;3.1)	2.4 (0.8;3.9) [i]	12.6 (10.6;14.5)	5.9 (4.5;7.3) [ii]	8.1 (6.5;9.8) [ii]	9.3 (7.4;11.2) [i]
GCSE D-G	3.0 (-0.5;6.4)	4.8 (1.6;8.0)	5.7 (2.5;8.9)	9.8 (6.2;13.5) [i]	-0.1 (-1.5;1.3)	1.0 (-1.0;3.0)	2.7 (-0.5;5.0) [i]	1.7 (-0.5;3.9)	23.6 (20.4;26.9)	12.2 (9.7;14.7) [ii]	13.8 (10.9;16.8) [ii]	14.5 (11.2;17.7) [ii]
No qualifications	1.8 (-1.4;5.0)	5.7 (2.8;8.7) [i]	8.3 (5.2;11.3) [ii]	12.9 (9.2;16.5) [ii]	1 (-0.3;2.3)	2.4 (0.7;4.1)	5.1 (3.0;7.1) [ii]	6.1 (3.4;8.9) [ii]	31.6 (28.3;34.8)	21.1 (18.2;24) [ii]	21.6 (18.4;24.0) [ii]	19.5 (15.9;23.1) [ii]

[ii] ≤0.001, [i] ≤0.05; significance test *p*-value for age PR differences (interaction) and PD differences (pairwise comparisons) (age 3 baseline).

Notes: Missing data (n) at age 3, 5, 7, 11 for: weight: 1373, 251, 340, 410; LLSI: 149, 82, 82,110; SED: 1164, 647, 492, 528; maternal academic attainment: 110, 100, 88, 69.
